# Adherence to the MIND dietary pattern and the association with cognitive outcomes in the UK Biobank

**DOI:** 10.1002/alz.087249

**Published:** 2025-01-09

**Authors:** Megan Smith, Sarah Bauermeister

**Affiliations:** ^1^ Dementias Platform UK ‐ University of Oxford, Oxford, Oxfordshire United Kingdom; ^2^ Dementias Platform UK ‐ University of Oxford, Oxford United Kingdom

## Abstract

**Background:**

Consumption of the Mediterranean‐Dietary Approaches to Stop Hypertension Diet Intervention for Neurodegenerative Delay (MIND) diet has been proposed to support advantageous cognitive outcomes. Intra Individual Variability (IIV) has been suggested as a useful outcome for predicting adverse cognitive outcomes. Executive function (EF) may be particularly susceptible to dietary influence. No studies have investigated the relationship between MIND adherence and IIV, nor MIND and EF in a large UK sample.

**Method:**

This was an observational study including 84,520 (56.1% female) participants from the UK Biobank Study who completed at‐least two 24‐hr dietary recalls and cognitive tests either at baseline or imaging visits. MIND diet adherence scores were derived using food intakes from multiple 24‐hr dietary recalls. IIV was constructed using the variability of response times within the reaction time task during baseline assessments. Executive function composite was created using path analysis, included tests were digit symbol substitution, trail making task ratio, fluid intelligence score and IIV. Multiple linear regressions were used to explore whether adhering to the MIND diet was associated with IIV or EF.

**Result:**

The mean (SD) MIND score was 6.8 (2.0) in the full sample. Adherence to the MIND diet was not associated with IIV. MIND dietary scores were significantly associated with worse performance in executive function tasks, albeit with a small beta value (β = ‐0.005, p = .003).

**Conclusion:**

Adhering to the MIND diet was not associated with advantageous cognitive outcomes in our analysis. Here, MIND diet was inversely related to performance in EF tasks, but no association found with IIV. The association with worse cognitive performance should be interpreted in the context of a small beta value and the likely influence of residual confounding. Moreover, as analysis was cross‐sectional, reverse causality cannot be ruled out. Differences between this analysis and prior literature in terms of location, sample size, dietary and cognitive assessments may also be contributing to the contradictory results. Further evaluation is needed to determine if MIND diet is associated with positive cognitive outcomes.

